# Warts

**Published:** 1891-08

**Authors:** 


### WARTS.

The cures for warts are exceedingly numerous. These which follow are given in the order of their ease and accessibility; but the needle plan, which comes nearly last, bears the palm for speed and certainty. In all cases where a solvent is employed, the hard, insensitive crown of the wart should be previously pared off, but not so deeply as to cause bleeding. And in most cases it is advisable to protect the adjacent skin either with a circle of vaseline or a piece of plaster with a hole in the middle which just fits the wart. Then apply as often as convenient, either the white juice of sow-thistle (sonchus oleraceus) which grows everywhere; or the yellow juice of greater celendine (chelidonium majus) which prefers the vicinity of human dwellings ; or the exceedingly corrosive, creamy sap of sun-spurge (euphorbia helioscopia), which likes gardens and cultivated fields. The next few remedies involve going to a chemist, namely, lunar caustic, once or twice a day, glacial acetic acid, salicylic acid and creosote, iodine and carbolic acid, or caustic potash, which is dangerous stuff. A piece of raw beef steeped twelve hours in vinegar and then held to the wart with rag or sticking-plaster is pronounced a sure cure in about a fortnight. The needle cure does its work in about ten minutes, of course, not counting the healing of the sore. It is done by running a darning-needle through the middle of the wart and holding the end of the needle in the flame of a candle. The heat is conducted along the steel so as to destroy the vitality of the wart, which is not so terrible a process as it sounds ; nevertheless, as it demands a little pluck, I have put it last on the list, and advise having some kind of forceps at hand to withdraw the hot needle if required.

